# Effect of Alcoholic Stimulants upon the Action of Anæsthetic Agents

**Published:** 1868-07

**Authors:** 


### EDITORIAL DEPARTMENT.

*Effect of Alcoholic Stimulants upon the Action of Anæsthetic Agents*—Several years ago our attention was called to the effect of such alcoholic stimulants as brandy and whiskey during the administration of chloroform to a patient who came into the office in a state of partial intoxicatiou. The rapidity with which the anæsthetic condition in this case was brought on, led to some experiments which convinced us that in the use of all general anæsthetics, the action of these agents is greatly facilitated by the previous administration of a small quantity of brandy or whiskey.

We have also tried the effects of such stimulants upon persons of very nervous temperaments with good results, and in no case was the slightest nausea produced.

Our attention has again been called to this subject by reading an article on the "Prevention of sickness from Chloroform," by Dr. J. R. Riley, in the "*Pacific Medical and Surgical Journal.* Dr. Riley after some remarks on the liability of chloroform to produce nausea and vomiting, and the use of stimulants to prevent such effects as these, says: that "the stimulating effect of the liquor furnishes a partial antidote to the dangerous influence of the terchloride in arresting the hearts action." "The patient recovers his sensibilities more rapidly and more completely, and in a great majority of cases is spared the troublesome nausea and vomiting which usually follow." "In this way" he says, "I believe its administration to be to a great extent perfectly safe, even in cases where there is reason to fear slight organic disease of the heart, or tubercular disease of the lungs." "I am in the habit of administering it in almost every operation with the knife or lancet, even also in extracting teeth, which I am sometimes called on to do in the absence of a dentist, but never without giving the brandy or whiskey first." "I can assure the profession that there is no harm in a trial of the two together and if they find that I am even partially correct, I shall be amply repaid for calling their attention to it."

